# Evaluation of photoreceptor-directed fibroblasts derived from retinitis pigmentosa patients with defects in the *EYS* gene: a possible cost-effective cellular model for mechanism-oriented drug

**DOI:** 10.1186/s13287-022-02827-x

**Published:** 2022-04-11

**Authors:** Dilip Rai, Masaki Iwanami, Yoriko Takahashi, Yukari Komuta, Noriyuki Aoi, Akihiro Umezawa, Yuko Seko

**Affiliations:** 1 Sensory Functions Section, Research Institute, National Rehabilitation Center for Persons With Disabilities, 4-1 Namiki, Tokorozawa, 359-8555 Japan; 2 Department of Ophthalmology, Hospital, National Rehabilitation Center for Persons With Disabilities, 4-1 Namiki, Tokorozawa, 359-8555 Japan; 3 Bioscience and Healthcare Engineering Division, Mitsui Knowledge Industry Co., Ltd., 2-7-14 Higashi-Nakano, Nakano-ku, Tokyo, 164-8555 Japan; 4 Department of Plastic, Oral and Maxillofacial Surgery, Teikyo University School of Medicine, 2-11-1 Kaga, Itabashi, Tokyo, 173-8605 Japan; 5 National Center for Child Health and Development, Research Institute, 2-10-1 Okura, Setagaya, 157-8535 Japan; 6 Present Address: Iwanami Eye Clinic, 7-1-3, Tsuchihashi, Miyamae-ku Kawasaki, Tokyo, 216-0005 Japan; 7 Present Address: Division of Bioinformation and Therapeutic Systems, National Defense Medical College, 3 Namiki, Tokorozawa, 359-0042 Japan; 8 Present Address: Miyamasuzaka Clinic, SK Aoyama Bldg. 5F, 1-6-5 Shibuya, Tokyo, 150-0002 Japan

**Keywords:** Redirect differentiation, Dermal fibroblast, Photoreceptor, Disease modeling, Retinitis pigmentosa, *EYS*, Semiquantitative analysis, *CRYGD*, *F2R*, ER stress, 4-PBA

## Abstract

**Background:**

The most common gene responsible for autosomal recessive retinitis pigmentosa (RP) is *EYS.* The manner of decay of genetically defective *EYS* gene transcripts varies depending on the type of mutation using our cellular model, which consists of induced photoreceptor-directed fibroblasts from EYS-RP patients (EYS-RP cells). However, disease-specific profiles have not been clarified in EYS-RP cells. Herein we investigated comprehensive gene expression patterns and restoration of altered expression by low molecular weight molecules in EYS-RP cells.

**Methods:**

Using induced photoreceptor-like cells by *CRX*, *RAX*, *NeuroD,* and *OTX2*, we employed qRT-PCR and DNA microarray analysis to compare expression levels of disease-related genes in EYS-RP cells. We investigated the effect of antiapoptotic or anti-endoplasmic reticulum (ER) stress/antioxidant reagents on the restoration of altered gene expression.

**Results:**

Expression levels of phototransduction-related genes (blue opsin, rhodopsin, S-antigen, GNAT1, GNAT2) were lower in EYS-RP cells. *CRYGD* was extracted by global gene expression analysis, as a downregulated, retina-related and apoptosis-, endoplasmic reticulum (ER) stress- or aging-related gene. Pathway enrichment analysis suggested that “complement and coagulation cascades,” “ECM-receptor interaction” and “PI3K-Akt signaling pathway” could be involved in EYS-RP-associated pathogenesis. Among the matching/overlapping genes involved in those pathways, *F2R* was suggested as an EYS-RP-associated gene. The downregulation of *CRYGD* and *F2R* was completely restored by additional 4-PBA, an inhibitor of ER stress, and partially restored by metformin or NAC. In addition, 4-PBA normalized the expression level of cleaved caspase-3.

**Conclusions:**

Our cellular model may reflect the ER stress-mediated degenerative retina and serve as a pathogenesis-oriented cost-effective rescue strategy for RP patients.

**Supplementary Information:**

The online version contains supplementary material available at 10.1186/s13287-022-02827-x.

## Background

Retinitis pigmentosa (RP) is an inherited retinal dystrophy. Defects in the *EYS* gene on chromosome 6q12 are a major cause of autosomal recessive (ar) RP [1–4]. In Japan, defects in the *EYS* gene such as c.4957dupA (p.Ser1653Lysfs*2) and c.8805C>A (p.Tyr2935*) have been identified as pathogenic mutations in about 20–30% of arRP patients [5–9]. Hereafter, arRP caused by defects in the *EYS* gene will be referred to as “EYS-RP.” EYS-RP is characterized as late onset and progressive in severity, leading to visual loss. Therefore, it is an urgent global issue to develop a strategy to delay progressive retinal dystrophy. Although gene therapy has been reported to be safe and effective for one type of inherited retinal dystrophy [10–13], and several drugs are promising [14–17], no established treatments are available for EYS-RP to date.

Because RP patients show heterogenous phenotypes due to heterogeneous gene defects, it may be ideal to determine inhibitory strategies depending on the specific pathogenesis. For this purpose, cellular models are available in place of human retinas. Induced photoreceptor cells derived from iPSCs of RP patients with defects of genes other than the *EYS* gene were reported to reproduce pathogenic phenotypes [18, 19], indicating that the number of rod photoreceptor cells was decreased and that endoplasmic reticulum (ER) stress might be involved. Although methods to generate photoreceptors from iPSCs have been established [20, 21], they are expensive and time-consuming. Another method, transduction of fate-determining transcription factors [22–25], which is called “redirect differentiation,” can produce photosensitive photoreceptor-like cells from somatic cells. Using “redirect differentiation,” we produced photoreceptor-like cells from dermal fibroblasts of EYS-RP patients with homozygous or heterozygous mutations, as a replacement for the degenerative retinas from EYS-RP patients [26]. In that study, we demonstrated that the manners of decay of the *EYS* gene transcripts varied, depending on type of defect in the *EYS* gene. It was also suggested that defects in the *EYS* gene might be relevant for cell growth rates and that the cell growth rate may be determined mainly by donor age. Therefore, findings in the EYS-RP-derived cells should be compared with those in age-matched control-derived cells for accurate assessment of the characteristics of EYS-RP-derived photoreceptor-like cells.

In the present study, we compared expression levels of phototransduction-related genes (blue opsin, rhodopsin, S-antigen, guanine nucleotide-binding protein G(t) subunit alpha-1 and 2 (GNAT1, GNAT2)) by qRT-PCR and gene expression profiles by microarray in photoreceptor-like cells from EYS-RP patients with those from age-matched normal volunteers. EYS-RP patients showed decreased levels of phototransduction-related genes, consistent with in vivo models reported previously [27]. By global gene expression profiling, CRYGD and F2Y were downregulated genes in EYS-RP-derived cells. We further investigated whether four drugs, 4-phenyl butyric acid (4-PBA), rapamycin, N-acetyl-L-cysteine (NAC), and metformin, could restore downregulation of these genes. Rapamycin, the inhibitor of mammalian target of rapamycin [28] (mTOR), has been suggested to inhibit photoreceptor degeneration in vivo [29, 30] and in vitro [31]. A well-known chemical chaperone, 4-PBA, is an FDA approved drug for urea cycle disorders owing to its effectiveness in reducing endoplasmic reticulum (ER) stress [32] and was reported to prevent photoreceptor degeneration [14]. The anti-inflammatory drug metformin had shown neuroprotective effect in *rd1* mice [17]. The antioxidant drug NAC suppressed photoreceptor death in *rd10* mice [15]. The present study showed that downregulation of phototransduction-related genes, CRYGD and F2R, in EYS-RP-derived cells was completely restored by 4-PBA, partially restored by metformin or NAC, but not by Rapamycin.

## Methods

### Isolation and culture of dermal fibroblasts

Dermal fibroblasts were harvested from three healthy donors [N#1 (53 years old), N#2 (48 years old), N#3 (63 years old)] and two EYS-RP patients (Pt#1, Pt#2, Table 1) under the approval of the Ethics Committee of the National Rehabilitation Center for Persons with Disabilities (NRCD). These five donors were studied in our previous paper [26]. Signed informed consent was obtained from the donors, and samples were de-identified. All experiments involving human cells and tissues were performed in line with the Declaration of Helsinki. Establishment of fibroblast culture derived from those five donors was performed as previously reported by us [26]. Seventh or eighth-passaged cells were used for following photoreceptor-directed differentiation.

**Table 1:** Defects in the EYS gene in patients for this study

Patient #	ID	Sex	Age (year)	Allele 1		Allele 2	
				Mutation	Effect	Mutation	Effect
1	RP38	F	67	c.4957dupA	p.Ser1653Lysfs*2	c.4957dupA	p.Ser1653Lysfs*2
2	RP174	M	58	c.4957dupA	p.Ser1653Lysfs*2	c.8805C>A	p.Tyr2935*

### Induction of photoreceptor-like cells (photoreceptor-directed fibroblasts)

Induction experiments were performed as previously reported [23, 24, 26]. In brief, full-length transcription factors, *CRX*, *RAX*, *NeuroD* and *OTX2*, were amplified from cDNAs prepared from total RNA of adult human retina (Clontech, CA, USA) by PCR and cloned into the XmnI-EcoRV sites of pENTR11 (Invitrogen). Preparation and infection with recombinant retrovirus were performed as previously reported [23]. In brief, the resulting pENTR11-transcription factors were recombined with pMXs-DEST by use of LR recombination reactions as instructed by the manufacturer (Invitrogen). The retroviral DNAs were then transduced into 293FT cells, and 3 days later the media were collected and concentrated. The human dermal fibroblasts were infected with this media containing retroviral vector particles as a mixture of the four transcription factors. After the retroviral infection, the media were replaced with the differentiation media, DMEM/F12/B27 medium supplemented with 40 ng/ml bFGF, 20 ng/ml EGF, fibronectin and 1% FBS. The retrovirus-infected cells were cultured for one to six weeks in 6-well or 24-well laminin-coated culture dishes. Expression of the four transgenes (*CRX, RAX, NeuroD* and *OTX2* (referred to as CRNO)) was confirmed by endpoint RT-PCR, and CRX was confirmed by immunocytochemistry in all photoreceptor-directed fibroblasts tested.

### Global gene expression analysis

To compare the gene expression profiles in photoreceptor-directed fibroblasts derived from EYS-RP patients with those from normal volunteers, we analyzed the expression levels of 58,201 probes in the induced/non-induced photoreceptor-like cells with or without EYS genes defects using the SurePrint G3 Human Gene Expression Microarray 8 × 60 K Ver.2.0 (Agilent Technologies, Palo Alto) using total RNA extracted from the cells. To average experimental variations, extracted total RNA samples were pooled into one tube from three independent induction experiments, and pooled samples were subjected to microarray analyses. To normalize the variations in staining intensity among chips, the 75th percentile of intensity distribution was aligned across arrays using GeneSpring software version 12.5 (Agilent). We first compared expression profiles for all the probes and then for refined probes based on GO terms: retina-related and apoptosis, oxidative stress, ER stress or aging (GO (retina) and GO (cell death), respectively). We extracted the intersection of two groups of genes, i.e., up- or downregulated and GO (retina) or GO (cell death)-related genes. For pathway enrichment analysis, WebGestalt2017 (http://www.webgestalt.org/) with KEGG pathway (Kyoto Encyclopedia of Genes and Genomes pathway) as a database was used.

### Drug administration

The dermal fibroblasts obtained from EYS-RP patients (Pt#1, Pt#2) and age-matched healthy individual (N#3, N#1) were seeded in laminin-coated (Biolamina, 521) 24-well plates. The fibroblasts were transduced with mixture of CRNO as described in the “Induction of photoreceptor-like cells (photoreceptor-directed fibroblasts)” section. After 5–6 h of retroviral transduction, the media in transduced fibroblast cells were replaced with differentiation media containing either one of the four drugs (rapamycin (Selleck chemicals, Cat#S1039), 4-PBA (WAKO, Cat#168-06471), NAC (WAKO, Cat#015-05132), metformin (Abcam, Cat#ab120847)), vehicle or no addition for Pt#1 and containing vehicle or no addition for N#3. Rapamycin was used at the final concentration of 10 nM from the stock solution of 1 M dissolved in 100% ethanol. The 4-PBA was used the concentration of 5 mM from the freshly prepared solution of 5 M 4-PBA in 100% ethanol. A final concentration of 5 mM metformin was prepared from the stock solution of 100 mM metformin in water. Freshly prepared 1 M NAC in water was further diluted to make a final concentration of 5 mM NAC. The media with or without drugs or vehicle was replaced thrice weekly. Finally, the cells were harvested 2 weeks post-transduction. Total RNA was isolated using a Picopure™ RNA isolation kit (Thermo Fisher Scientific; Cat#KIT0204) following the manufacturer’s protocol. cDNAs were synthesized from total RNA using superscript™ IV first-strand synthesis system (Thermo Fisher Scientific; Cat#18091050).

### Reverse transcriptase (RT)-PCR

Total RNA was isolated with RNeasy Plus mini kit® (Qiagen, Maryland, USA) or PicoPure™ RNA Isolation Kit (Arcturus Bioscience, CA, USA) according to the manufacturer's instructions. An aliquot of total RNA was reverse-transcribed by using an oligo (dT) primer. A cDNA template was amplified using the Platinum Quantitative PCR SuperMix-UDG with ROX (Invitrogen) and ABI7900HT Sequence Detection System (Applied Biosystems). Fluorescence was monitored during every PCR cycle at the annealing step. The authenticity and size of the PCR products were confirmed using a melting curve analysis (using software provided by Applied Biosystems) and a gel analysis. mRNA levels were normalized using β-actin as a housekeeping gene. The expression level in the photoreceptor-directed fibroblasts, HDF-a (Human Dermal Fibroblasts-adult, ScienCell Research Laboratories), 2 weeks post-transduction was used as a reference. The design of the PCR primer sets is shown in our previous paper [23] and Table S2 (Additional file 2).

### Immunocytochemistry

The cells were seeded in laminin-coated 24-well plate (Sumilon MS-92132). The induction of photoreceptor-like cells was carried out mentioned above. The cells were fixed with 4% paraformaldehyde in 1 × PBS 14 days after transduction. The cells were washed and permeabilized with 0.1% Triton in 1 × PBS. The cells were incubated in blocking buffer (5% normal goat serum in 1 × PBS) for an hour at room temperature. The cells incubated with primary antibody (anti-CRX (1:1000, Abnova H00001406-M02), anti-rhodopsin (1D4) (1:200, Santa Cruz biotechnology, sc-57432)) in blocking buffer for overnight at 4C. Then, cells were incubated with secondary antibody (goat anti-mouse IgG, alexa flour® 568 conjugate (1:500, Invitrogen, A-11004)). The nuclei were stained with 4',6-diamidino-2-phenylindole (DAPI). Images were captured with a Nikon Eclipse TE300, and contrast and brightness were adjusted with NIS elements AR 3.2.

### Western blots

The induced photoreceptor-directed fibroblasts were lysed with radioimmunoprecipitation assay (RIPA) buffer (50 mM tris pH 8.0, 150 mM NaCl, 0.1% sodium dodecyl sulfate, 0.5% sodium deoxycholate and 1% NP40 substitute) containing 1X protease inhibitor cocktail (Roche). The cell lysates were homogenized with gentle agitation for 30 min on ice and centrifuged at 12,000 rpm at 4 °C for 20 min. Each supernatant was collected in a new microcentrifuge tube. The protein concentration of each sample was measured by the Pierce™ BCA Protein Assay Kit (ThermoScientific). The samples were denatured with 4 X Laemlli sample buffer (Biorad), heated at 95 °C for 5 min, resolved in 4–20% gel (Biorad) and transferred to PDVF membrane (Biorad). Detection of immune complexes formed by proteins of interest and primary antibodies (anti-blue opsin (1:250, Milipore, AB5407), anti-CHOP (1:1500, Cell Signaling Technology, #2895), anti-cleaved caspase-3 (1:1000, Cell signaling technology, #9664), anti-β-actin (1:1000, Cell Signaling Technology)) was performed by enzyme-linked color development with anti-mouse horseradish peroxidase (HRP)-conjugated secondary antibody (1:3000, Cell Signaling Technology). The signals based on chemical luminescence (ECL™ Prime Western Blotting system (Amersham)) were detected by ChemiStage (TOYOBO). The band intensity was quantified with ImageJ software (National Institutes of Health).

### Statistical analysis

Analysis of variance (ANOVA) with Tukey’s honest test was employed to compare of the gene expression level of multiple groups with the photoreceptor-directed fibroblasts derived from the healthy individuals (control group). *p* value less than 0.05 was considered significant.

## Results

### Expression levels of phototransduction-related genes were lower in induced photoreceptor-like cells from fibroblasts of EYS-RP patients

**Fig. 1:**
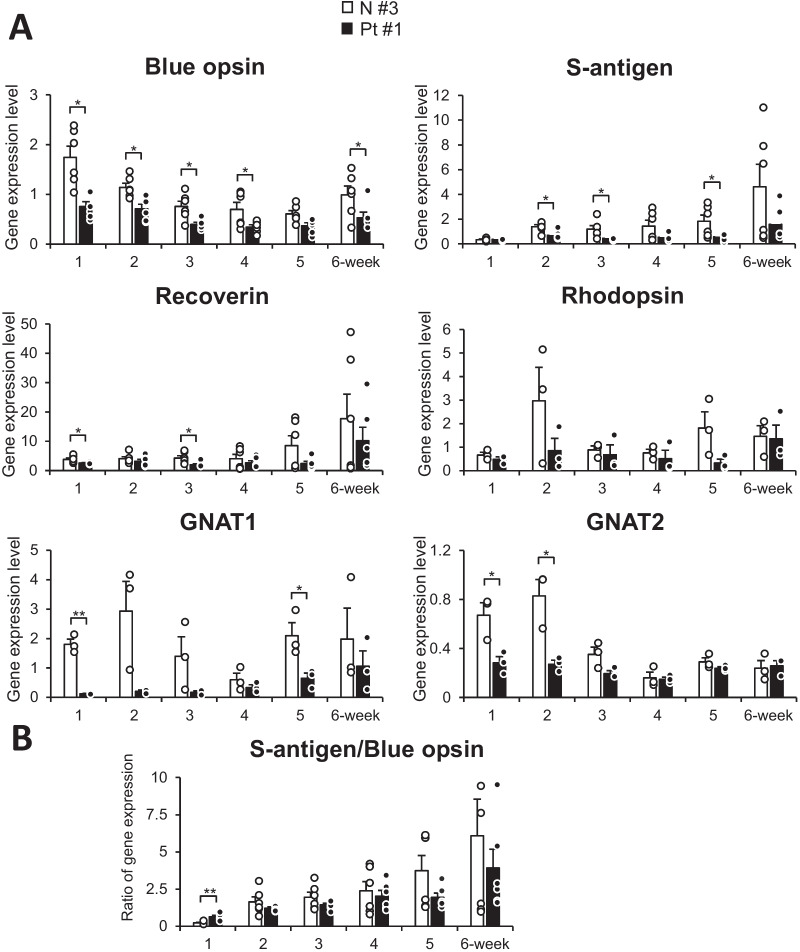
Induction of retina-specific genes in human dermal fibroblasts derived from a healthy individual (N#3, open box) and an RP patient (Pt#1, closed box) by retroviral infection of genes for transcription factors CRX, RAX, NeuroD and OTX2. **A** RT-PCR analysis for phototransduction-related genes, blue opsin, S-antigen, rhodopsin, GNAT1, GNAT2, recoverin, in cultured human dermal fibroblasts (HDF). Vertical axis; relative expression, Horizontal axis; weeks after transduction. Columns represent mean ± SEM. All data points are overlaid. **p < 0.01; student’s t-test (*n* = 6 (blue opsin, S-antigen, recoverin), *n* = 3 (GNAT1, GNAT2). **B** Ratio of expression levels of rod photoreceptor- and cone photoreceptor-specific genes in photoreceptor-like cells (S-antigen vs blue opsin). Error bar: SEM. **p < 0.01; student’s t-test (*n* = 6)

It was previously reported that immunostaining of blue opsin was decreased in EYS-deficient zebrafish [33]. We compared expression levels of phototransduction-related genes, *OPN1SW* (the blue opsin gene), *SAG* (the S-antigen gene), *RCVRN* (the recoverin gene), *RHO* (the rhodopsin gene), *GNAT1* and *GNAT2*. From one to six weeks after CNRO gene transduction, expression levels of the blue opsin gene, the recoverin gene and the GNAT1 gene were lower in photoreceptor-directed fibroblasts derived from EYS-RP patients (Fig. 1). In EYS-RP cells, the lower expression levels of the S-antigen gene initiated 2 weeks after transduction and expression levels were significantly lower 2, 3 and 5 weeks after transduction. Expression levels of the GNAT2 gene were significantly lower one and two weeks after transduction in EYS-RP cells. The expression levels of the rhodopsin gene were lower in EYS-RP cells from one to six weeks, though not significantly (Fig. 1). Expression of blue opsin, rhodopsin and CRX were also detected by immunocytochemistry in photoreceptor-directed fibroblasts derived from both normal healthy volunteers and EYS-RP patients (Additional file 2: Fig. S1, Figure S3 in our previous paper [26]); therefore, statistical analysis was performed on western blots for blue opsin (Additional file 2: Fig. S4).

### *NRL*, a key transcription factor for rod differentiation, was downregulated gene in the photoreceptor-directed fibroblasts derived from EYS-RP patients

**Fig. 2:**
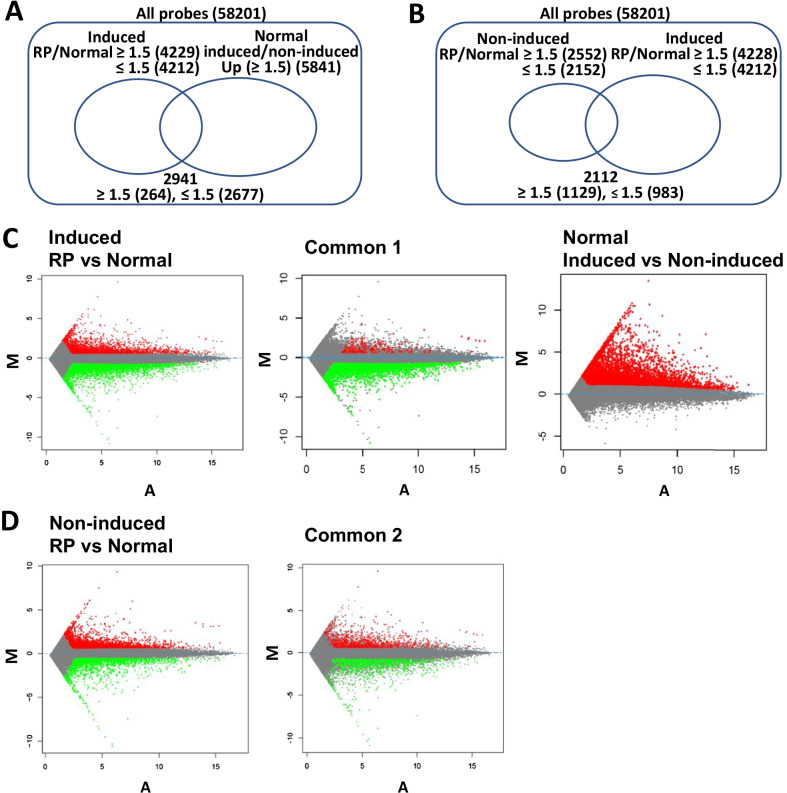
Categorization of genes that are differentially expressed in photoreceptor-directed fibroblasts derived from an EYS-RP patient and upregulated by transduction of *CRX*, *RAX*, *NeuroD* and *OTX2*. Venn diagrams (**A**, **B**) and Mean-average (MA) plots (**C**, **D**) to compare EYS-RP cells vs normal cells. To clarify the specific gene expression profile in induced photoreceptor cells derived from EYS-RP patients, we compared the expression profiles of 58,201 probes in the induced photoreceptor cells or non-induced fibroblast cells derived from an EYS-RP patient (induced or non-induced, RP, Pt#1) and healthy volunteers (induced or non-induced, normal, N#3), using microarrays. We first extracted the intersection of the two groups of genes, i.e., up- or downregulated genes in RP versus healthy ([induced Pt#1] versus [induced N#3], which is an age-matched pair for comparison) (signal ratio ≥  + 1.5, ≤ -1.5 for “up,” “down”) and those in CRNO-transduced normal fibroblasts versus non-induced fibroblasts ([induced N#3] versus [non-induced N#3]) (signal ratio ≥  + 1.5) (**A**). Furthermore, we compared non-induced EYS-RP-derived cells and non-induced normal cells, and then extracted the intersection of the two groups to define up- or downregulated genes (non-induced Pt#1] versus [non-induced N#3]). We also determined up- or downregulated genes in induced RP versus induced healthy ([induced Pt#1] versus [induced N#3]) (signal ratio ≥  + 1.5, ≤ -1.5) (**B**). Mean-average (MA) plots indicate differential gene expression between induced RP and induced normal (**C**) and differential gene expression between non-induced RP and non-induced normal (**D**). Significantly differentially upregulated genes (signal ratio ≥  + 1.5) are highlighted in red and downregulated genes (signal ratio ≤ -1.5) are highlighted in n green

To clarify the specific gene expression profiles in induced photoreceptor cells derived from EYS-RP patients, we compared the expression profiles of 58,201 probes in the induced photoreceptor cells derived from EYS-RP patients (induced, RP) and healthy volunteers (induced, normal). We first extracted the intersection of the two groups of genes, i.e., up- or downregulated genes in RP versus healthy ([induced Pt#1] versus [induced N#3], which is an age-matched pair for comparison) (signal ratio ≥  + 1.5, ≤ -1.5 for “up,” “down”) and those in CRNO-transduced normal fibroblasts versus non-induced fibroblasts ([induced N#3] versus [non-induced N#3]) (signal ratio ≥  + 1.5). A total of 264 upregulated genes and 2677 downregulated genes were extracted, which suggest that there are more downregulated genes in the photoreceptor-directed fibroblasts derived from EYS-RP patients compared to those from normal controls (Fig. 2). Among the 2677 downregulated genes, 50 genes were significantly decreased in another independently performed microarray analysis using independently differentiated photoreceptor-directed fibroblasts (Pt#1 vs N#2), which is an age-unmatched pair (Table 2). Among the genes whose expression levels were greatly decreased, *NRL*, a key transcription factor for rod differentiation, was extracted. We then compared non-induced EYS-RP-derived cells and non-induced normal cells. It was shown that there were more differentially expressed genes, EYS-RP vs normal, in the differentiated experimental groups (4229 upregulated probes, 4212 downregulated probes) (Fig. 2A) than in the non-induced groups (2552 upregulated probes, 2152 downregulated probes) (Fig. 2B).

**Table 2:** Downregulated genes in EYS-RP-derived photoreceptor-like cells

GeneSymbol	GeneName	Pt #1 non-induced (1)	Pt #1 induced (1)	N #3 non-induced (1)	N #3 induced (1)	Pt #1 non-induced (2)	Pt #1 induced (2)	N #2 non-induced (2)	N #2 induced (2)
LZTS1	Leucine zipper, putative tumor suppressor 1	3.24	5.45	5.39	9.43	2.81	5.24	2.98	6.85
SST	Somatostatin	1.00	4.40	1.11	8.27	2.89	4.29	2.27	8.05
TNNC1	Troponin C type 1 (slow)	3.42	4.27	3.68	7.76	2.70	4.39	2.98	7.01
FOXS1	Forkhead box S1	1.66	3.84	1.76	7.07	1.07	4.16	1.94	6.00
ANO2	Anoctamin 2, calcium activated chloride channel	1.48	4.51	2.48	7.60	1.78	4.58	1.86	6.31
HES6	Hes family bHLH transcription factor 6	2.03	4.08	2.28	7.15	3.11	5.29	3.23	6.47
NLRP12	NLR family, pyrin domain containing 12	0.79	3.12	0.93	6.10	1.19	3.17	0.29	6.42
KRT18	Keratin 18, type I	1.63	4.02	4.34	6.90	2.40	3.67	3.05	6.57
HES6	Hes family bHLH transcription factor 6	7.15	10.31	7.33	13.15	7.54	10.83	8.04	12.32
CKMT1A	Creatine kinase, mitochondrial 1A	1.11	6.16	1.17	8.82	1.67	5.82	1.42	7.10
		3.06	3.93	3.94	6.52	2.75	3.79	3.05	5.96
DIRAS3	DIRAS family, GTP-binding RAS-like 3	4.08	7.61	5.35	10.11	4.10	8.07	4.62	9.89
PDLIM3	PDZ and LIM domain 3	2.93	5.31	4.28	7.78	0.59	4.16	0.50	5.70
C8orf46	Chromosome 8 open reading frame 46	2.37	5.04	2.69	7.47	1.08	5.38	1.02	7.36
ASMT	Acetylserotonin O-methyltransferase	3.27	3.88	3.05	6.28	1.92	4.36	1.47	7.99
COL4A1	Collagen, type IV, alpha 1	5.41	7.26	6.24	9.66	6.17	7.64	6.19	9.59
LYPD1	LY6/PLAUR domain containing 1	4.31	5.37	5.84	7.73	4.45	5.13	3.44	7.71
C2CD4A	C2 calcium-dependent domain containing 4A	0.52	6.08	0.51	8.36	1.21	5.85	2.08	7.60
**NRL**	**Neural retina leucine zipper**	**3.15**	**8.05**	**2.60**	**10.31**	**2.86**	**8.76**	**1.91**	**10.17**
COL4A2	Collagen, type IV, alpha 2	8.89	10.43	9.70	12.66	9.76	10.68	9.49	12.02
**NRL**	**Neural retina leucine zipper**	**4.04**	**8.84**	**4.12**	**11.06**	**3.83**	**9.51**	**3.46**	**11.02**
LINC00463	Long intergenic non-protein coding RNA 463	0.84	4.19	0.96	6.34	0.85	4.35	0.45	6.09
ADRA2A	Adrenoceptor alpha 2A	4.64	7.20	4.12	9.27	4.29	7.40	3.74	8.79
NTRK3	Neurotrophic tyrosine kinase, receptor, type 3	2.36	2.14	0.59	4.06	1.04	3.06	1.54	5.67
NGFR	Nerve growth factor receptor	0.55	4.51	0.60	6.43	0.55	4.91	0.50	7.60
MPPED2	Metallophosphoesterase domain containing 2	0.65	5.29	0.82	7.18	0.37	5.64	0.33	7.84
AMER2	APC membrane recruitment protein 2	0.78	8.01	0.80	9.76	0.68	7.97	1.49	9.80
CTSH	Cathepsin H	6.82	7.64	7.24	9.36	6.46	7.30	6.02	9.51
PDZD2	PDZ domain containing 2	2.19	6.21	2.38	7.92	0.39	6.33	0.99	8.45
TCF15	Transcription factor 15 (basic helix-loop-helix)	6.12	5.76	6.57	7.45	5.49	5.57	5.53	6.92
F2R	Coagulation factor II (thrombin) receptor	2.38	4.05	2.99	5.68	3.10	4.80	3.53	6.61
GRAMD1C	GRAM domain containing 1C	5.88	7.06	6.06	8.62	4.88	7.00	4.46	8.98
GPX3	Glutathione peroxidase 3 (plasma)	4.82	8.34	5.16	9.87	4.60	8.44	3.98	10.67
BEGAIN	Brain-enriched guanylate kinase-associated	4.33	4.39	5.47	5.92	4.45	4.63	4.82	6.59
TMEM176A	Transmembrane protein 176A	3.66	9.80	3.69	11.31	4.13	10.15	4.75	11.75
A2M	Alpha-2-macroglobulin	7.24	5.96	8.35	7.45	8.67	6.78	9.16	8.00
TMSB15A	Thymosin beta 15a	4.54	4.05	5.37	5.51	3.62	4.14	4.51	5.85
GPRC5B	G protein-coupled receptor, class C, group 5, member B	3.92	7.32	3.45	8.77	4.13	7.61	3.75	10.06
IMPG1	Interphotoreceptor matrix proteoglycan 1	0.60	4.33	0.56	5.74	0.54	3.93	0.43	6.05
PLEKHB1	Pleckstrin homology domain containing, family B (evectins) member 1	4.54	7.65	4.43	9.05	4.63	7.46	3.97	9.67
SYPL2	Synaptophysin-like 2	7.12	7.76	6.99	9.15	6.21	7.72	5.26	9.92
SLC16A3	Solute carrier family 16 (monocarboxylate transporter), member 3	11.28	11.44	13.32	12.77	13.11	11.60	14.03	12.88
CBX2	Chromobox homolog 2	2.08	5.02	1.71	6.21	3.61	5.17	3.52	6.36
CCDC13	Coiled-coil domain containing 13	2.07	6.52	2.39	7.66	1.83	7.00	1.92	8.75
MECOM	MDS1 and EVI1 complex locus	4.27	3.93	4.27	5.06	3.67	3.40	4.43	5.65
RXRG	Retinoid X receptor, gamma	0.69	6.30	0.72	7.40	0.42	6.87	0.39	8.48
CCDC181	Coiled-coil domain containing 181	3.07	8.39	3.76	9.48	3.08	8.35	3.84	9.70
GNB3	Guanine nucleotide binding protein (G protein), beta polypeptide 3	3.90	6.99	3.99	8.06	3.62	7.18	4.42	8.70
OXTR	Oxytocin receptor	6.26	7.83	5.87	8.86	4.82	7.44	4.35	9.65
		4.79	4.45	5.12	5.46	3.95	4.72	4.92	6.30

### *CRYGD* was downregulated in EYS-RP-derived cells, retina-related and apoptosis/oxidative stress/ER stress/aging-related genes

**Fig. 3:**
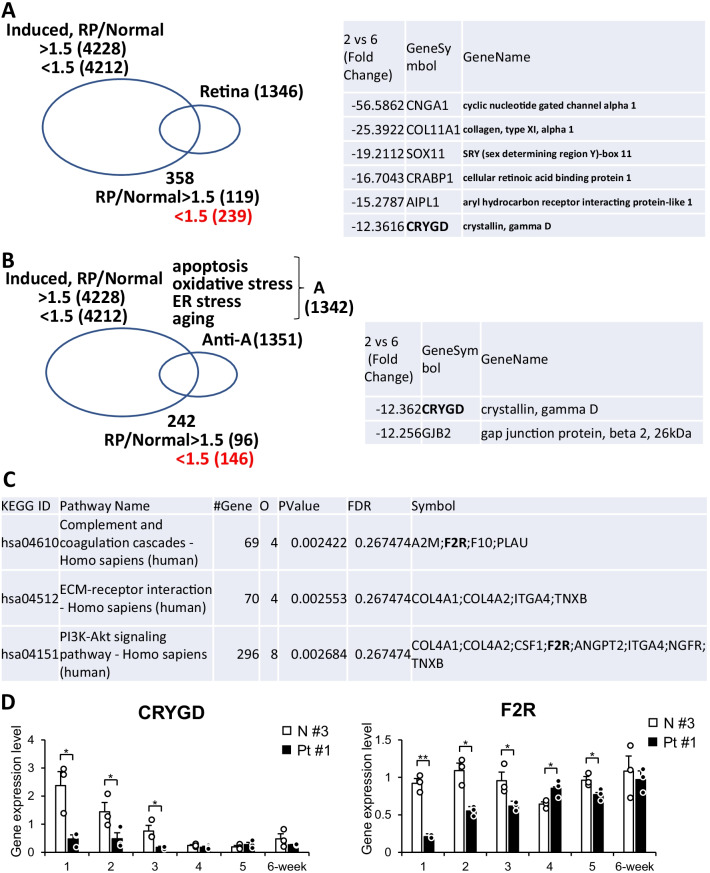
Global gene expression analysis of EYS-RP patient-derived photoreceptor-like cells. **A** Categorization of genes differentially expressed in both photoreceptor-directed fibroblasts derived from an EYS-RP patient and retina-related genes. We extracted intersection of the two groups of genes, i.e., up- or downregulated genes in RP versus healthy and retina-related genes according to gene ontology (GO) term annotation, which was used in our previous study [24]. A total of 119 upregulated genes and 239 downregulated genes were extracted (left panel). *CNGA1*, *COL11A1*, *SOX11*, *AIPL1* and *CRYGD* showed significant decreases in expression (Right panel). **B** Categorization of genes that are both differentially expressed in photoreceptor-directed fibroblasts derived from an EYS-RP patient and apoptosis/oxidative stress/ER stress/aging-related genes according to gene ontology (GO) term annotation. Ninety-six upregulated genes and 146 downregulated genes were extracted, with CRYGD and GJB2 displaying large decreases. **C** EYS-RP-associated pathway extracted by pathway enrichment analysis. Pathway enrichment analysis was performed on a gene set that is an intersection of up- or downregulated genes in RP versus age-matched healthy ([induced Pt#1] versus [induced N#3], and another intersection of up- or downregulated genes in RP versus age-unmatched healthy ([induced Pt#1] versus [induced N#2], which was served for the analysis. By pathway enrichment analysis, WebGestalt2017 (http://www.webgestalt.org/), three pathways, “complement and coagulation cascades,” “ECM-receptor interaction” and “PI3K-Akt signaling pathway,” were extracted from up- or downregulated genes in photoreceptor-directed fibroblasts derived from EYS-RP compared to normal volunteers. Among the matching/overlapping genes in the three pathways, the F2R gene was involved in both the “complement and coagulation cascades” and “PI3K-Akt signaling pathway.” **D** RT-PCR analysis of expression of *CRYGD* and *F2R* genes in photoreceptor-directed fibroblasts derived from an EYS-RP patient (Pt#1) and a normal volunteer (N#3). The vertical axis is relative expression, and the horizontal axis is weeks after gene transduction. Decreased expression of *CRYGD* and *F2R* in EYS-RP-derived cells was confirmed. The expression levels in photoreceptor-directed fibroblasts transduced with four transcription factor genes (*CRX*, *RAX*, *NeuroD* and *OTX2*) was calculated from 3 biological replicates. Columns represent mean ± SEM. All data points are overlaid. *p<0.05, **p<0.01; student’s t-test. Internal control: β-actin.

We then extracted intersection of the two groups of genes, i.e., up- or downregulated genes in RP versus healthy and retina-related genes with gene ontology (GO) term annotation, which was used in our previous study [24]. A total of 119 upregulated genes and 239 downregulated genes were extracted, which suggests that there are downregulated genes in RP retinas (Fig. 3). *CNGA1*, *COL11A1*, *SOX11*, *AIPL1* and *CRYGD* showing the largest decreases in expression. We then extracted the interaction of the two groups of genes, i.e., up- or downregulated genes in RP versus healthy and apoptosis/oxidative stress/ER stress/aging-related genes according to gene ontology (GO) term annotation. Those four key words were chosen based on previous studies of animal models of RP [34–37] and the fact that EYS-RP is late-onset and progressive disease. Ninety-six upregulated genes and 146 downregulated genes were extracted, with *CRYGD* and *GJB2* showed the largest decreases in expression. Decreased expression of *CRYGD* in EYS-RP-derived cells was confirmed by qRT-PCR.

### F2R may be involved in an EYS-RP-specific pathway

We then prepared a gene set for pathway enrichment analysis, an intersection of up- or downregulated genes in RP versus age-matched healthy ([induced Pt#1] versus [induced N#3], and another intersection of up- or downregulated genes in RP versus age-unmatched healthy ([induced Pt#1] versus [induced N#2]. Using WebGestalt, we found that three pathways, “complement and coagulation cascades,” “ECM-receptor interaction” and “PI3K-Akt signaling pathway,” were extracted from up- or downregulated genes in photoreceptor-directed fibroblasts derived from EYS-RP compared to normal volunteers. Among the matching/overlapping genes in the three pathways, the F2R gene was found in both “complement and coagulation cascades” and “PI3K-Akt signaling pathway.” Decreased expression of F2R in EYS-RP-derived cells was confirmed by qRT-PCR.

## Decreased expression levels of the phototransduction-related genes, *OPN1SW* (blue opsin), *RCVRN* (recoverin), *GNAT1* and *GNAT2*, and the genes extracted by global gene expression analysis, *GRYGD *and *F2R*, in EYS-RP-derived cells were increased by the chemical chaperone 4-PBA

**Fig. 4:**
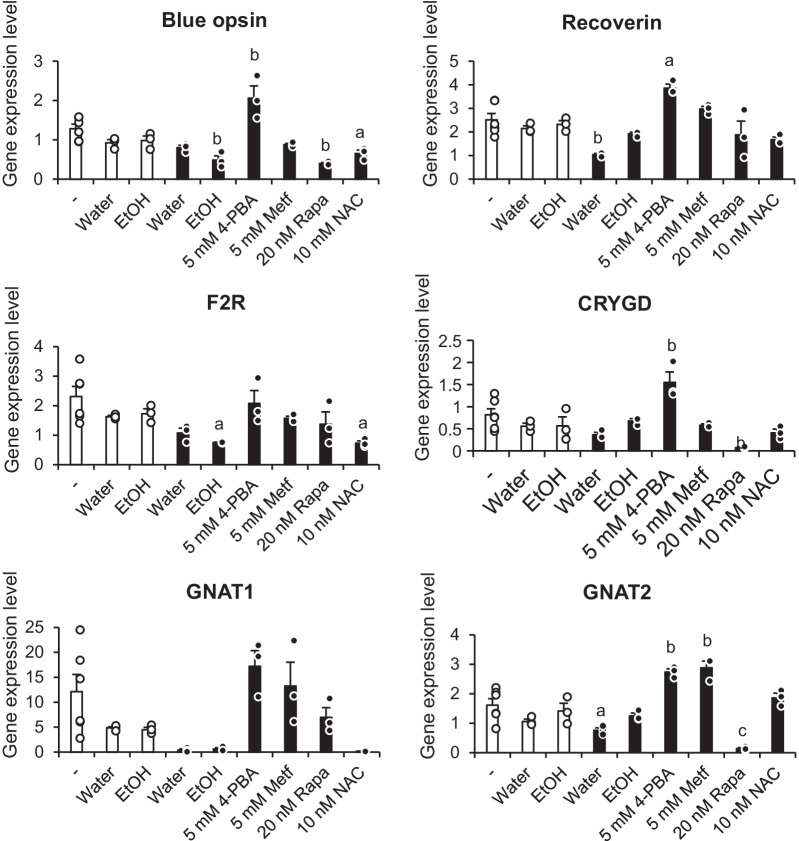
Effect of drugs on gene expression in photoreceptor-directed fibroblasts derived from an EYS-RP patient, Pt#1. The fibroblasts of a healthy individual (N#3) and fibroblasts of a patient suffering from retinitis pigmentosa due to a homozygous mutation in the *EYS* gene (Pt#1) were transdifferentiated to photoreceptor-like cells by retroviral transduction of four transcription factors. Gene expression was compared 2 weeks post-transduction. Differentiation medium was supplemented with four drugs, 4-phenylbutyric acid (4-PBA), metformin (Metf), rapamycin (Rapa), N-acetyl-L-cysteine (NAC) or the vehicle (water and ethanol (EtOH)) or none (-). The gene expression with each pharmacological treatment was compared to the gene expression in photoreceptor-like cells derived from fibroblasts of healthy individual without any supplement (-). Columns represent mean ± SEM. All data points are overlaid (*n* = 6 (N#3 without any supplement (-)), *n* = 3 (N#3 with supplement and Pt#1). a = *p* > 0.05, b = *p* > 0.01, c = *p* > 0.001; One-way ANOVA followed by Tukey’s honest test. For the sake of simplicity, comparison with the gene expression level in N#3 without any supplement (-) is shown here. Comparison among other groups is shown in Additional file 2: Fig. S2

We tested whether drugs would increase levels of gene expression in photoreceptor-directed fibroblasts derived from EYS-RP to similar levels of those from normal volunteers. Six target genes whose expression levels were decreased in EYS-RP derived cells were chosen (blue opsin, recoverin, GNAT1, GNAT2, F2R and CRYGD), based on previous results in the present study. The effectiveness of four drugs was evaluated. 4-PBA was found to be the most effective. The treatment of photoreceptor-directed fibroblasts derived from Pt#1 with 5 mM 4-PBA restored the expression levels of target genes to levels similar to the control group, or higher than the control (N#3) (Fig. 4, Additional file 2: Fig. S2). The gene expression levels with 4-PBA treatment were significantly higher than those of photoreceptor-like cells with a vehicle, EtOH (one-way ANOVA followed by Tukey’s test). The restoration effect by 4-PBA treatment was confirmed by western blotting for the blue opsin (Additional file 2). Similarly, three doses of 4-PBA were tested to determine whether expression levels of the target genes in photoreceptor-directed fibroblasts derived from Pt#2 were also restored to levels of the control (N#1) (Additional file 2: Fig. S3). The application of metformin also increased the expression levels of target genes to comparable or higher levels than the control. However, metformin was less effective in restoring the expression of target genes in photoreceptor-directed fibroblasts derived from Pt#2 (Fig. S3). This discrepancy in effectiveness of metformin might be due to the difference in the point mutation in *EYS* between Pt#1 and Pt#2; however, more samples are needed to clarify this discrepancy. Application of rapamycin and NAC did not restore the expression level of any target genes in photoreceptor-directed fibroblasts derived from Pt#1 to similar levels of the control (N#3) (Fig. 4), as well as Pt#2 versus N#1 (Fig. S3). The expression levels of these genes in photoreceptor-like cells derived from fibroblast of Pt#1 treated with vehicle (water or ethanol) were lower than in control group, however, not significantly (Fig. 4, one-way ANOVA followed by Tukey’s test). A similar trend in reduction in the expression level of target genes was also observed in photoreceptor-directed fibroblasts derived from Pt#2, with a heterozygous mutation in the *EYS* gene, and an aged-matched healthy individual (N#1) (Fig. S3).

Based on the above results with 4-PBA, we investigated UPR-signal transduction in EYS-deficient cellular models. Inositol requiring enzyme 1α/β (IRE1) is the one of the key UPR signal activator proteins [38–40]. Thus, we examined the relative expression of *ERN* (endoplasmic reticulum to nucleus signaling), which codes for IRE1. We found that the relative expression of *ERN* was significantly lower in photoreceptor-directed fibroblasts derived from Pt#2 and was also lower in that from Pt#1, although not significantly (Additional file 2: Fig. S4). Treatment with 5 mM 4-PBA restored the relative expression levels of *ERN* in photoreceptor-directed fibroblasts derived from both patients to similar levels a found in healthy individuals (Fig. S4). CHOP (C/EBP homologous protein) is one of the target genes of the UPR pathway and has a role in controlling gene expression that leads to apoptosis [38–40]. We examined the expression level of CHOP by western blotting, using photoreceptor-directed fibroblasts derived from Pt#1 and N#3. CHOP was undetectable in both fibroblasts. Upon treatment with thapsigargin (200 mM), an ER stress inducer, the expression levels of CHOP were slightly higher in Pt#1-derived cells than N#3, albeit statistically insignificant (Fig. S4). We performed western blotting with a cleaved caspase-3 antibody to investigate the restoration effect of 4-PBA on apoptosis. The relative expression of cleaved caspase-3 was slightly higher in EYS-RP than photoreceptor-like cells derived from a healthy individual, although there was no significant difference. The relative expression of cleaved caspase-3 was clearly decreased by 4-PBA treatment. By immunostaining, caspase-3 was also detected in more photoreceptor-directed fibroblasts derived from Pt#1 rather than N#3 under thapsigargin supplementation (Additional file 2: Fig. S5). These results indicate that EYR-RP-derived cells are more sensitive to an ER stress inducer.

## Discussion

This is the first report that shows gene expression profiles of cellular models of EYS-RP patients. Because our previous study suggested that findings of patient-derived cells should be compared with age-matched normal volunteer to assess EYS-RP-associated characteristics [26], we here compared gene expression profiles in induced photoreceptor-directed fibroblasts derived from EYS-RP patients with age-matched normal volunteers. As a result, expression levels of several phototransduction-related genes, blue opsin, recoverin, GNAT1, GNAT2, and an aging-related gene, *CRYGD*, and complement and coagulation cascades- and PI3K-Akt signaling pathway-related gene, *F2R,* were significantly lower in photoreceptor-directed fibroblasts derived from EYS-RP patients compared to age-matched normal volunteers. This decrease was reversed by addition of 4-PBA and metformin, but not by rapamycin or NAC.

One of the downregulated genes, the γD-crystallin encoding CRYGD gene, which was extracted as both retina-related and apoptosis/oxidative stress/ER stress/aging-related genes (Fig. 3), is also known as a cataractogenesis-related gene [41]. On the other hand, SLC16A10 (LAT2), which is also a cataractogenesis-related gene [42], was extracted by pathway enrichment analysis as involved in the “protein digestion and absorption pathway” (Additional file 1: Table S1), in the intersection of up- or downregulated genes in RP versus age-matched healthy volunteer and those in CRNO-transduced normal fibroblasts versus non-induced fibroblasts. RP, most likely including EYS-RP, is a typical late-onset retinal degenerative disease, with cataract formation noted at relatively younger ages [43–45]. The mechanism for pathologic, not physiological, aging-related cataractogenesis in EYS-RP has not been elucidated; however, it may be explained, at least in part, by downregulation of the *CRYGD* gene in photoreceptors of EYS-RP patients. Furthermore, crystallin plays roles outside of the lens, with cilia-related functions in photoreceptors [46]. Of note, defects in human γD-crystallin, known to form amyloid aggregates, were suggested to play a role in retinal pigment epithelial cells, leading to age-related macular degeneration [47]. Our results suggest possible roles of downregulated γD-crystallin in EYS-associated photoreceptor degeneration as well as cataractogenesis. In the present study, Gap junction protein, beta 2 (*GJB2*) showed decreased expression by the same analysis. The *GJD2* gene, which encodes connexin 36, was reported to be related to myopia development [48]. It is also known that *GJD2* plays a role in retinal signal transduction and is expressed in gap junctions between cones, rods and bipolar cells, playing essential roles in rod-mediated visual signals [49, 50]. Further studies are needed to clarify the molecular interactions of CRYGD and GJD2 with EYS in the retina.

EYS, an agrin/perlecan-related extracellular matrix protein secreted by photoreceptors into the interrhabdomeral space [51], was thought to be comparable to interphotoreceptor or subretinal space of the human retina. A recent report supports the possible function of EYS as an extracellular matrix protein in the retina of a zebrafish model with defects in the *eys* gene [52]. From our present study using a human cellar model of EYS-RP, interphotoreceptor matrix proteoglycan 1 (IMPG1) (Table 2) and ECM-receptor interaction pathway (Fig. 3, Table S1) were extracted as an EYS-RP-associated gene and pathway, suggesting the ECM-related functions of EYS throughout evolution.

On the other hand, in previous reports using zebrafish, the EYS protein is localized near connecting cilium/transition zone in photoreceptors [27, 32, 53] and disruption of the ciliary pocket in cones is observed in *eys*^−/−^ [32]. The study further showed that expression of opsin, localized in outer segment of photoreceptors, is extremely lower in *eys*^−/−^, and the authors speculate that the lower expression is due to disruption of transport in cilia. We here show that expression levels of several phototransduction-related genes, blue opsin, recoverin, GNAT1, GNAT2 were significantly lower in photoreceptor-directed fibroblasts derived from EYS-RP patients compared to age-matched normal volunteers. A regulatory mechanism other than transportation via connecting cilium may exist in EYS deficiency because our photoreceptor-like cells lack outer segments, although gathering mitochondria were observed [23]. On the other hand, Nrl, a key molecule to control rod-photoreceptor development [54, 55], was extracted as one of the significantly downregulated genes in EYS-RP derived cells in this study (Table 2). This may lead to the typical phenotype of RP or EYS-RP, rod-cone dystrophy [56, 57], although atypical phenotypes of RP, such as cone/cone-rod dystrophy and sector RP, have been reported [58–60]. It is also noteworthy that expression levels of blue opsin and the ratio of expression levels of blue opsin vs S-antigen were significantly lower in the earlier days after CRNO gene transduction in EYS-RP-derived photoreceptor-like cells, which may be explained by the fact that up-regulation of cone-photoreceptor-related genes, started earlier than rod-photoreceptor genes [23], which are consistent with previous reports on retinogenesis. In our redirect differentiation study, it is difficult to discern whether downregulation of phototransduction-related genes is related to delay, defects of retinogenesis or decrease of differentiation efficiency in EYS-RP cells. Correlation of gene expression profiles to phenotype should be helpful, as well as genotype–phenotype relation for personalized medicine.

Pathway enrichment analysis revealed that three pathways were extracted from differentially expressed genes between EYS-RP and normal volunteers (Fig. 3). In those pathways, *F2R*, which encodes protease-activated receptor-1 (PAR-1), was matching/overlapping in both “complement and coagulation cascades” and “PI3K-Akt signaling pathway.” Decreased expression of the *F2R* gene in EYS-RP-derived cells was confirmed by qRT-PCR (Fig. 3). Those pathways and the *F2R* gene may be promising as therapeutic targets because there is evidence of the involvement of inflammation in the pathogenesis of RP [61–64]. F2R may also be involved in prevention of apoptosis [65], morphogenesis of photoreceptors [66] and survival of cancer cells through a Bcl-xL-dependent mechanism [67]. Reduction of *F2R* expression by defects in the EYS gene may be relevant to multiple cascades in EYS-RP.

It was reported that a defect in the rhodopsin gene, P23H, the most common cause of RP in the USA, induces rhodopsin misfolding and the unfolded protein response (UPR) [36]. Also, it was shown that the endoplasmic reticulum (ER)-UPR, a known mechanism of apoptosis secondary to an overwhelming accumulation of misfolded protein [68], is involved in photoreceptor degeneration caused by missense mutations in TULP1 [69], which are associated with two forms of IRDs, early-onset retinitis pigmentosa (RP) and Leber congenital amaurosis (LCA). In this study, we demonstrated that 4-phenylbutyric acid (4-PBA), chemical chaperone, an ER stress inhibitor, dramatically restored downregulation of phototransduction-related genes, *CRYGD* and *F2R*, suggesting that ER stress may be a possible therapeutic target for photoreceptor degeneration in EYS-RP patients.

In our previous paper, we demonstrated that the defective *EYS* mRNA containing c.4957dupA in photoreceptor-directed fibroblasts of Pt#1 and Pt#2 undergoes partial degradation [26]. In the present study, we show that the expression level of *ERN*, which codes IRE1, was decreased in photoreceptor-directed fibroblasts derived from Pt#1 and Pt#2. It is well established that IRE1 degrades mRNA via RIDD (regulated Ire1-dependent decay) pathway to mitigate ER stress [38–40]. The failure to degrade faulty mRNA in EYS-RP might be attributable to lowered expression of ERN. IRE1 has also been reported to promote secretion of Spacemaker/Eyes shut in the intrarhabdomeral space in Drosophila [70]. This could be one of the reasons for depletion of IRE1 expression. Additionally, IRE1 is reported to play a role in photoreceptor differentiation via RIDD pathway [70]. We speculate the decreased level of IRE1 might be due to the reduced protein translation in response to ER stress. It is plausible that the depletion of IRE1 might have affected the redirect differentiation in EYS-RP, thereby causing a decrease in expression levels of phototransduction-related genes. We are planning additional detailed studies on mRNA degradation by the RIDD pathway to clarify the abovementioned hypothesis. Our study also showed that the 4-PBA effectively restored the expression levels of *ERN* in EYS-RP-derived induced cells to levels similar of healthy individuals. Apparently, 4-PBA may attenuate the ER stress by various mechanisms. One plausible mechanism of 4-PBA-mediated upregulation of several EYS-RP-associated genes might be due to upregulation of *ERN*, thereby possibly promoting mRNA degradation of defective EYS mRNA or facilitating the redirect differentiation to photoreceptors. To clarify this speculation, further studies are needed.

By our pathway enrichment analysis using microarray data of EYS-RP-patient-derived photoreceptor-like cells, “hypertrophic cardiomyopathy (FDR: 0.011684)” and “dilated cardiomyopathy (FDR: 0.014503)” were extracted (Table S1). It was suggested that EYS protein in Drosophila plays a role in protecting mechanoreceptor and chemoreceptor organs from hyperosmotic shock by providing stiffness and maintaining cellular integrity and tissue morphogenesis [71]. A genetic study suggested an association between trastuzumab-induced cardiotoxicity and rs139944387 in exon 44 of Eyes shut homologs [EYS] [72]. On the other hand, in Drosophila, localization and function of actomyosin machinery in photoreceptors were previously demonstrated [73]. The mitochondria-related pathway may also be a common mechanism between photoreceptors and cardiomyocytes, considering the fact that both cell types include highly dense mitochondria in the cytosol. Creatinine kinase, mitochondrial 1A was also extracted as a differentially expressed gene (Table 2). Taken together, these results suggest that defects of *EYS* are involved in a common pathway between photoreceptor degeneration and dysfunction of cardiomyocytes; however, further studies are needed to confirm this hypothesis.

Although iPSC lines were established from patients with defects in RHO [31], MAK [74], PDE6A [75], PRPF31 [76], NR2E3 [77] and EYS [78], molecular mimicry of the iPSC-derived retina to the degenerative retina in human RP has not been confirmed. A high-throughput assay system for drug screening using human cellular models is necessary, although zebrafish model could complement such an assay [79]. The present study suggests that our cellular model may be promising for such a screening system. Our system, however, has the limitation that our photoreceptor-like cells lack other surrounding cell types, such as tissue-resident microglial cells [80] and infiltrating cells such as monocytes and lymphocytes [81], which are relevant to pathogenesis of RP. That may be why an antioxidant, NAC, did not restore decreased expression of *CRYGD* or *F2R*. However, our photoreceptor-like cells derived from EYS-RP should be a helpful cellular model, considering that rod-photoreceptor cells are primarily injured by the genetic mutations. Another limitation is that 4-PBA, which was found to restore downregulation of several EYS-associated genes, may not restore other unknown EYS-associated changes. We are planning to set up single-cell analysis to overcome this limitation. Our cellular model can be improved by using as yet undiscovered exogenous factors in the differentiation media. Furthermore, we will have to verify the present findings using other models such as iPSC-derived retinas or in vivo models in the future.

## Conclusions

In summary, we here compared gene expression profiles of photoreceptor-like cells derived from dermal fibroblasts of EYS-RP patients with those of age-matched healthy donors to determine the molecular mechanism of photoreceptor degeneration in EYS-RP patients. The photoreceptor-like cells were produced by redirect differentiation with four transcription factors, CRX, RAX, NeuroD and OTX2, as a cellular model. By global gene expression analysis, several retina-related and apoptosis/oxidative stress/ER stress/aging-related genes such as opsin gene, CRYGD and F2R were differentially expressed in EYS-RP patient-derived cells. Decreased expression levels were restored by addition of a chemical chaperone, 4-PBA. These results suggest that retinal degeneration of EYS-RP is due, at least in part, to ER stress and that the redirect differentiation method could be a valuable tool for disease model despite some limitations as a replacement for the degenerative retinas.

## Supplementary Information

**Additional file 1: Table S1.** EYS-RP-associated pathways.

**Additional file 2 Table S2.** Primer list. **Figure S1.** Immunostaining of CRX and rhodopsin in photoreceptor-like cells derived from fibroblasts of a healthy individual (N#3) and EYS-RP (Pt#1) after 2 weeks of induction. Scale bar = 50 μm. **Figure S2.** Comparison of the effects of pharmacological treatment on gene expression among groups, N#3 and Pt#1, with or without supplement. The same graph is shown as in Fig. 4 with other analytical results. For the sake of simplicity, comparison with the gene expression level in N#3 without any supplement (-) is shown in Fig. 4. Comparison among other groups is shown here. Column represents mean ± SEM (*n* = 6 (N#3 without any supplement (-)), *n* = 3 (N#3 with supplement and Pt#1). a = *p* > 0.05, b = *p* > 0.01, c = *p* > 0.001; One-way ANOVA followed by Tukey’s honest test. **Figure S3.** Effect of drugs on gene expression in photoreceptor-directed fibroblasts derived from an EYS-RP patient, Pt#2. The fibroblast cells obtained from patients suffering from retinitis pigmentosa with heterozygous mutations in *EYS* gene (Pt#2) and an age-matched healthy individual (N#1) were transduced with mixture of retroviral vectors encoding CRX, RAX, NeuroD and OTX2. Gene expression was compared 2 weeks post-transduction. The differentiation media were supplemented with four drugs namely, 4-phenylbutyric acid (4-PBA; 2 mM, 5 mM and 10 mM), metformin (METF; 5 mM), rapamycin (Rapa; 4 nM, 10 nM, 20 nM, 50 nM and 100 nM), N-acetyl-L-cysteine (NAC; 10 mM) or the vehicle (EtOH) or no addition (-). The gene expression levels with different pharmacological treatments were compared to those of photoreceptor-like cells derived from a normal volunteer (N#1) without any drug or vehicle. a = *p* > 0.05, b = *p* > 0.01, c = *p* > 0.001; One-way ANOVA followed by Dunnett’s test. **Figure S4.** A. Immunoblot of blue opsin and β-actin in HDF-a as negative control, photoreceptor-like cells derived from a healthy individual (N#3), EYS-RP (Pt#1) supplemented with 5 mM 4-PBA (Pt#1 + 4PBA) (panel a) and relative expression of the blue opsin (panel b). The band intensity of blue opsin was normalized by β-actin. Data represent mean + SEM (*n* = 3). *p < 0.05, **p < 0.01; Oneway ANOVA followed by Tukey’s test. B. Effect of 4-PBA in relative expression of *ERN* in EYS-RP. (a) Relative *ERN*1 expression in photoreceptor-like cells derived from N#3 with/out vehicle (EtOH) and RP#1 supplemented with 5 mM 4-PBA or vehicle. (b) Relative *ERN1* expression in photoreceptor-like cells derived from Fib#1 with/out vehicle (EtOH) and RP#3 supplemented with 5 mM 4-PBA or vehicle. *p > 0.05; One-way ANOVA followed by Tukey’s test (*n* = 3). C. a) Immunoblot of CHOP and β–actin of photoreceptor-like cells derived from fibroblasts of N#3 and RP#1 with 0 nM, 200 nM and 2 μM thapsigargin. b) Relative expression of CHOP in photoreceptor-like cells derived from fibroblasts of N#3 and RP#1 without or without thapsigargin with 0 nM, 200 nM and 2 μM thapsigargin. Student’s t-test (*n* = 3 (0), *n* = 5 (200 nM), *n* = 5 (2 μM)). D. Immunoblot of cleaved caspase-3 and β-actin in HDF-a treated with 2 μM staurosporine for 4 h before harvesting (HDF-a + Staruo) as positive control, photoreceptor-like cells derived from a healthy individual (N#3) and EYS-RP patient (Pt#1) supplemented with 5 mM 4-PBA (Pt#1 + 4PBA) (panel a) and expression level of the cleaved caspase-3 (panel b). The band intensity of cleaved caspase-3 was normalized by β-actin. Data represent mean + SEM (*n* = 3). *p < 0.05, **p < 0.01; Oneway ANOVA followed by Tukey’s test. **Figure S5.** Immunostaining of cleaved caspase-3 and numbers of positively stained cells in photoreceptor-directed fibroblasts of healthy individual (N#3) and EYS-RP (Pt#1) 2 weeks post-transduction supplemented by thapsigargin (1 µM). Cells were fixed in chilled acetone at -20 °C for 20 min followed by rinsing and incubated with 4% PFA in PBS for 10 min. After washing and blocking, cells were incubated with primary antibody (cleaved caspase-3 (Asp175) (5A1E) Rabbit mAb #9664, 1: 500, Cell Signaling).

## Data Availability

All data generated or analyzed for this study are included in this published article and the Additional file. Microarray data are deposited in the GEO with the accession number GSE199050.

## Abbreviations

RP: Retinitis pigmentosa
arRP: Autosomal recessive retinitis pigmentosa
EYS-RP: ArRP with defects in the *EY**S* gene
4-PBA: 4-Phenyl butyric acid
UPR: Unfolded protein response
CHOP: C/EBP homologous protein
