# Supplementary material for: Evaluation of photoreceptor-directed fibroblasts derived from retinitis pigmentosa patients with defects in the EYS gene: a possible cost-effective cellular model for mechanism-oriented drug
Source: Stem Cell Res Ther. 2022 Apr 11;13:157. doi: 10.1186/s13287-022-02827-x (PMC8996485; doi:10.1186/s13287-022-02827-x)
Supplement: Supplementary file 1 — Additional file 1: Table S1. EYS-RP-associated pathways. [file 13287_2022_2827_MOESM1_ESM.pdf]

Table S1 EYS-RP--associated pathways

KEGG ID	Pathway Name	#Gene	PValue	FDR
hsa04974	Protein digestion and absorption - Homo sapiens (human)	22	4.22E-06	0.001261
hsa00534	Glycosaminoglycan biosynthesis - heparan sulfate / heparin - Homo sapiens (human)	10	1.93E-05	0.00288
hsa05410	Hypertrophic cardiomyopathy (HCM) - Homo sapiens (human)	19	0.000117	0.011684
hsa04514	Cell adhesion molecules (CAMs) - Homo sapiens (human)	27	0.000176	0.013165
hsa05414	Dilated cardiomyopathy - Homo sapiens (human)	19	0.000243	0.014503
hsa04350	TGF-beta signaling pathway - Homo sapiens (human)	17	0.000548	0.027315
hsa04068	FoxO signaling pathway - Homo sapiens (human)	23	0.001181	0.050445
hsa04360	Axon guidance - Homo sapiens (human)	28	0.001545	0.052487
hsa04390	Hippo signaling pathway - Homo sapiens (human)	25	0.00158	0.052487
hsa04080	Neuroactive ligand-receptor interaction - Homo sapiens (human)	39	0.002289	0.068427
hsa04512	ECM-receptor interaction - Homo sapiens (human)	15	0.003199	0.086948
hsa05412	Arrhythmogenic right ventricular cardiomyopathy (ARVC) - Homo sapiens (human)	14	0.004128	0.102849
hsa04060	Cytokine-cytokine receptor interaction - Homo sapiens (human)	35	0.008387	0.192897
hsa04022	cGMP-PKG signaling pathway - Homo sapiens (human)	24	0.01097	0.224689
hsa04670	Leukocyte transendothelial migration - Homo sapiens (human)	18	0.011272	0.224689
hsa04015	Rap1 signaling pathway - Homo sapiens (human)	29	0.013564	0.253472
hsa05144	Malaria - Homo sapiens (human)	9	0.014952	0.262983
hsa04260	Cardiac muscle contraction - Homo sapiens (human)	13	0.017368	0.288502
hsa04918	Thyroid hormone synthesis - Homo sapiens (human)	12	0.025297	0.398019
hsa04020	Calcium signaling pathway - Homo sapiens (human)	24	0.026664	0.398019
hsa04971	Gastric acid secretion - Homo sapiens (human)	12	0.028227	0.398019
hsa04024	cAMP signaling pathway - Homo sapiens (human)	26	0.029286	0.398019
hsa00601	Glycosphingolipid biosynthesis - lacto and neolacto series - Homo sapiens (human)	6	0.032747	0.42023
hsa00604	Glycosphingolipid biosynthesis - ganglio series - Homo sapiens (human)	4	0.033731	0.42023
hsa00512	Mucin type O-glycan biosynthesis - Homo sapiens (human)	6	0.039109	0.430086
hsa05323	Rheumatoid arthritis - Homo sapiens (human)	13	0.039682	0.430086
hsa04750	Inflammatory mediator regulation of TRP channels - Homo sapiens (human)	14	0.040445	0.430086
hsa05020	Prion diseases - Homo sapiens (human)	7	0.040792	0.430086
hsa04964	Proximal tubule bicarbonate reclamation - Homo sapiens (human)	5	0.042515	0.430086
hsa04550	Signaling pathways regulating pluripotency of stem cells - Homo sapiens (human)	19	0.043152	0.430086
hsa04530	Tight junction - Homo sapiens (human)	18	0.046914	0.452491
hsa04261	Adrenergic signaling in cardiomyocytes - Homo sapiens (human)	19	0.052841	0.45701
hsa04014	Ras signaling pathway - Homo sapiens (human)	27	0.053508	0.45701
hsa04510	Focal adhesion - Homo sapiens (human)	25	0.054006	0.45701
hsa05206	MicroRNAs in cancer - Homo sapiens (human)	20	0.055126	0.45701
hsa04330	Notch signaling pathway - Homo sapiens (human)	8	0.059298	0.45701
hsa04151	PI3K-Akt signaling pathway - Homo sapiens (human)	38	0.060003	0.45701
hsa04933	AGE-RAGE signaling pathway in diabetic complications - Homo sapiens (human)	14	0.060915	0.45701
hsa04911	Insulin secretion - Homo sapiens (human)	12	0.060953	0.45701
hsa04972	Pancreatic secretion - Homo sapiens (human)	13	0.061138	0.45701
hsa04270	Vascular smooth muscle contraction - Homo sapiens (human)	15	0.064937	0.473564
hsa00380	Tryptophan metabolism - Homo sapiens (human)	7	0.069744	0.496513
