# Supplementary material for: Evaluation of photoreceptor-directed fibroblasts derived from retinitis pigmentosa patients with defects in the EYS gene: a possible cost-effective cellular model for mechanism-oriented drug
Source: Stem Cell Res Ther. 2022 Apr 11;13:157. doi: 10.1186/s13287-022-02827-x (PMC8996485; doi:10.1186/s13287-022-02827-x)
Supplement: Supplementary file 2 — Additional file 2 Table S2. Primer list. Figure S1. Immunostaining of CRX and rhodopsin in photoreceptor-like cells derived from fibroblasts of a healthy individual (N#3) and EYS-RP (Pt#1) after 2 weeks of induction. Scale bar = 50 μm. Figure S2. Comparison of the effects of pharmacological treatment on gene expression among groups, N#3 and Pt#1, with or without supplement. The same graph is shown as in Fig. 4 with other analytical results. For the sake of simplicity, comparison with the gene expression level in N#3 without any supplement (-) is shown in Fig. 4. Comparison among other groups is shown here. Column represents mean ± SEM (n = 6 (N#3 without any supplement (-)), n = 3 (N#3 with supplement and Pt#1). a = p > 0.05, b = p > 0.01, c = p > 0.001; One-way ANOVA followed by Tukey’s honest test. Figure S3. Effect of drugs on gene expression in photoreceptor-directed fibroblasts derived from an EYS-RP patient, Pt#2. The fibroblast cells obtained from patients suffering from retinitis pigmentosa with heterozygous mutations in EYS gene (Pt#2) and an age-matched healthy individual (N#1) were transduced with mixture of retroviral vectors encoding CRX, RAX, NeuroD and OTX2. Gene expression was compared 2 weeks post-transduction. The differentiation media were supplemented with four drugs namely, 4-phenylbutyric acid (4-PBA; 2 mM, 5 mM and 10 mM), metformin (METF; 5 mM), rapamycin (Rapa; 4 nM, 10 nM, 20 nM, 50 nM and 100 nM), N-acetyl-L-cysteine (NAC; 10 mM) or the vehicle (EtOH) or no addition (-). The gene expression levels with different pharmacological treatments were compared to those of photoreceptor-like cells derived from a normal volunteer (N#1) without any drug or vehicle. a = p > 0.05, b = p > 0.01, c = p > 0.001; One-way ANOVA followed by Dunnett’s test. Figure S4. A. Immunoblot of blue opsin and β-actin in HDF-a as negative control, photoreceptor-like cells derived from a healthy individual (N#3), EYS-RP (Pt#1) supplemented with 5 mM 4-PBA (Pt#1 + 4PBA) ( [file 13287_2022_2827_MOESM2_ESM.pdf]

Table S2: Primer list

Gene	Forward (5' → 3')	Reverse (5' → 3')
Rhodopsin	ACAGGATGCAATTTGGAGGGC	GCTCATGGGCTTACACACCA
GNAT1	CACTCAACATCCAGTACGGAGA	CCTCCTCGATAGTGTCTGCC
GNAT2	CACCAGAAGAATGCCTGGAGT	AGCCAGGTTGTTGAGCTGTC
CRYGD	ACCGCTTCCGCTTCAATGAAA	CGTCCTCGGTAGTTGGACA
F2R	GCCATCGTTGTGTTCATCCTG	GCTGATCTTAAAGGGGAGCAC
ERN	CACAGTGACGCTTCCTGAAAC	GCCATCATTAGGATCTGGGAGA
β-actin	CATGTACGTTGCTATCCAGGC	CTCCTTAATGTCACGCACGAT

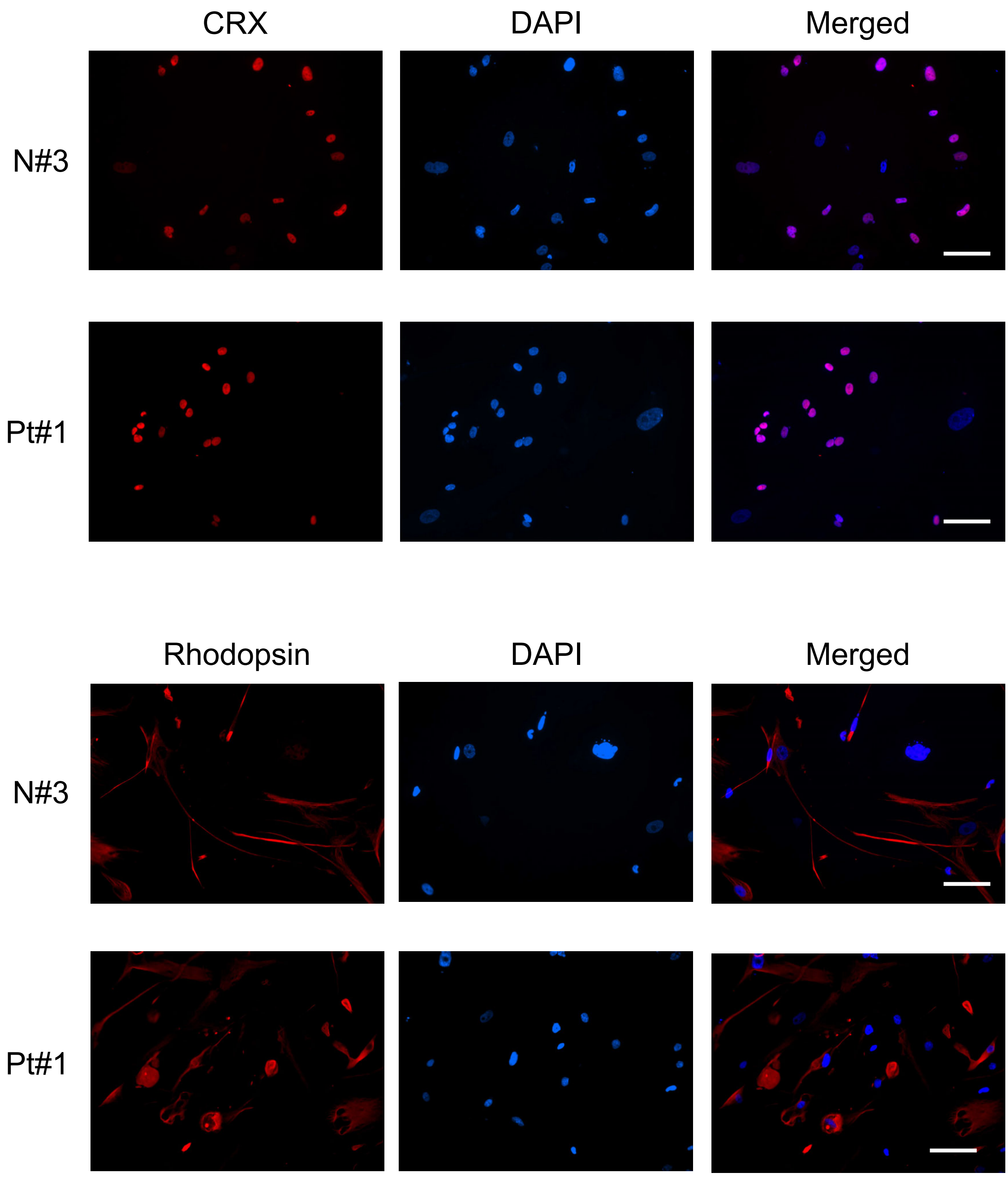

Figure S1

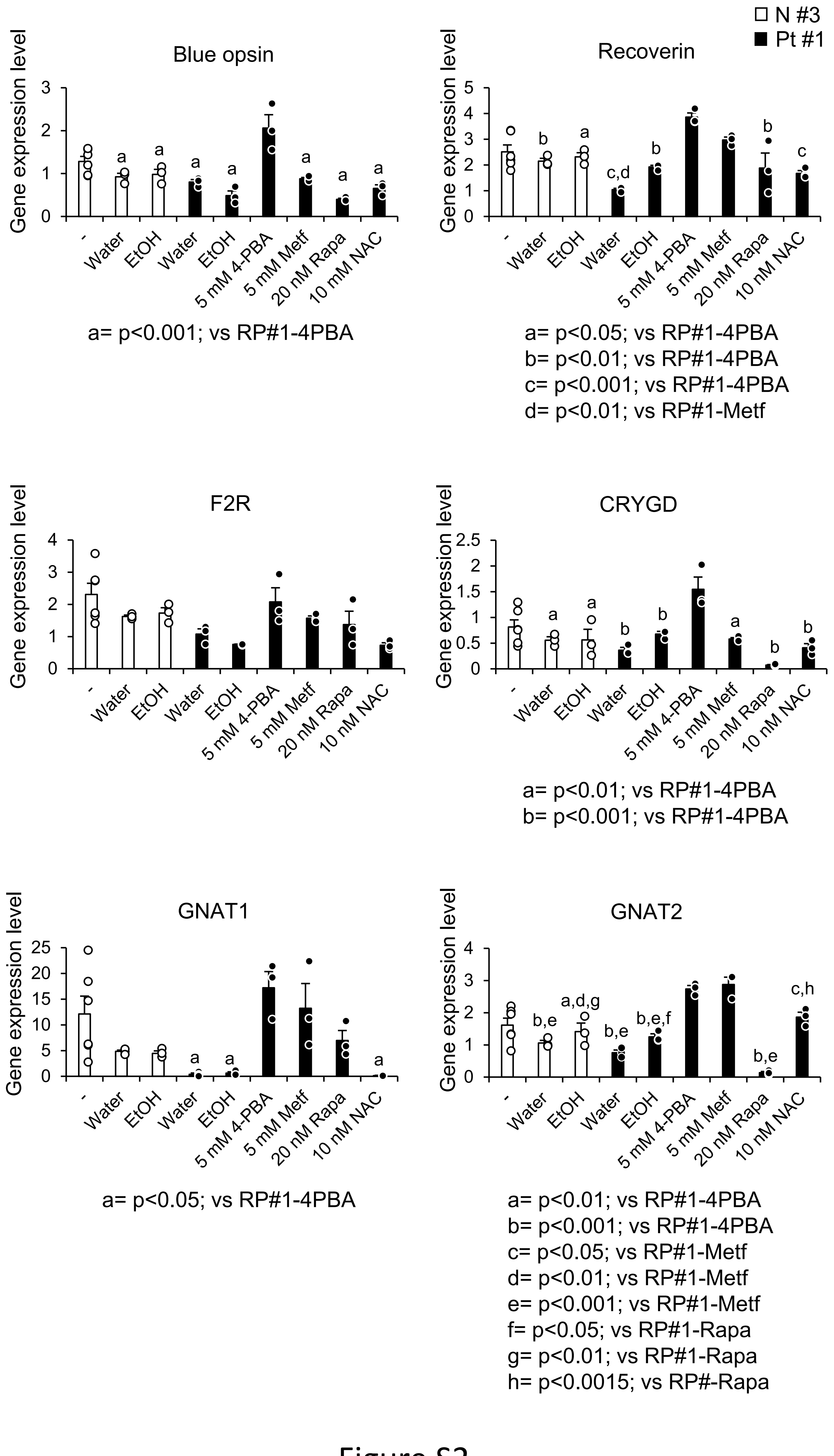
N #3
Pt #1
Blue opsin
Gene expression level
-
Water
EtOH
Water
EtOH
5 mM 4-PBA
5 mM Metf
20 nM Rapa
10 mM NAC
a= p<0.001; vs RP#1-4PBA
Recoverin
a= p<0.05; vs RP#1-4PBA
b= p<0.01; vs RP#1-4PBA
c= p<0.001; vs RP#1-4PBA
d= p<0.01; vs RP#1-Metf
F2R
CRYGD
a= p<0.01; vs RP#1-4PBA
b= p<0.001; vs RP#1-4PBA
GNAT1
a= p<0.05; vs RP#1-4PBA
GNAT2
a= p<0.01; vs RP#1-4PBA
b= p<0.001; vs RP#1-4PBA
c= p<0.05; vs RP#1-Metf
d= p<0.01; vs RP#1-Metf
e= p<0.001; vs RP#1-Metf
f= p<0.05; vs RP#1-Rapa
g= p<0.01; vs RP#1-Rapa
h= p<0.0015; vs RP#-Rapa

Figure S2

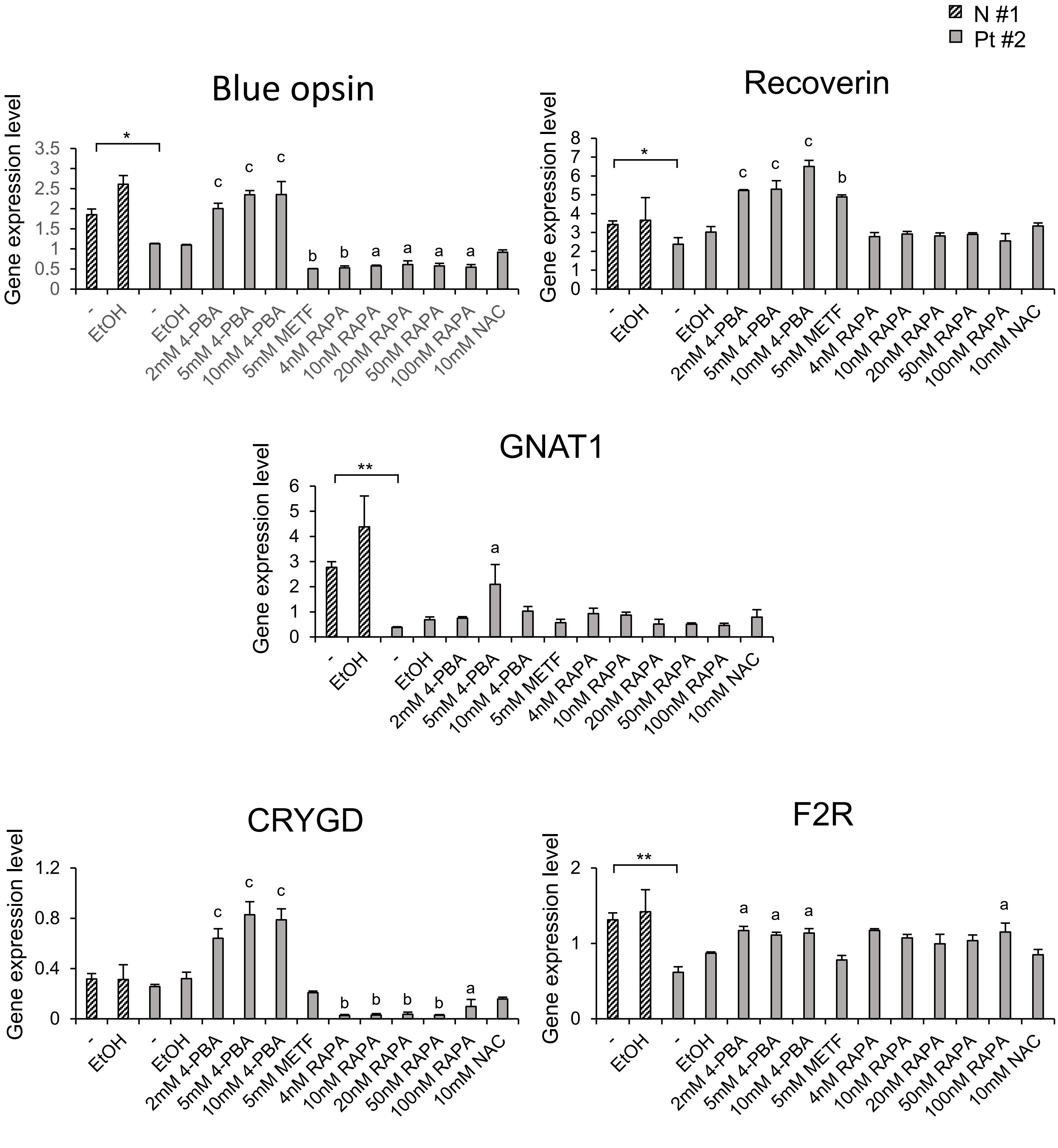
N #1
Pt #2
Blue opsin
Gene expression level
3.5
3
2.5
2
1.5
1
0.5
0
*
c
c
c
b
b
a
a
a
a
-
EtOH
-
EtOH
2mM 4-PBA
5mM 4-PBA
10mM 4-PBA
5mM METF
4nM RAPA
10nM RAPA
20nM RAPA
50nM RAPA
100nM RAPA
10mM NAC
Recoverin
Gene expression level
8
7
6
5
4
3
2
1
0
*
c
c
c
b
GNAT1
Gene expression level
6
5
4
3
2
1
0
**
a
CRYGD
Gene expression level
1.2
0.8
0.4
0
c
c
c
b
b
b
b
a
F2R
Gene expression level
2
1
0
**
a
a
a
a

Figure S3

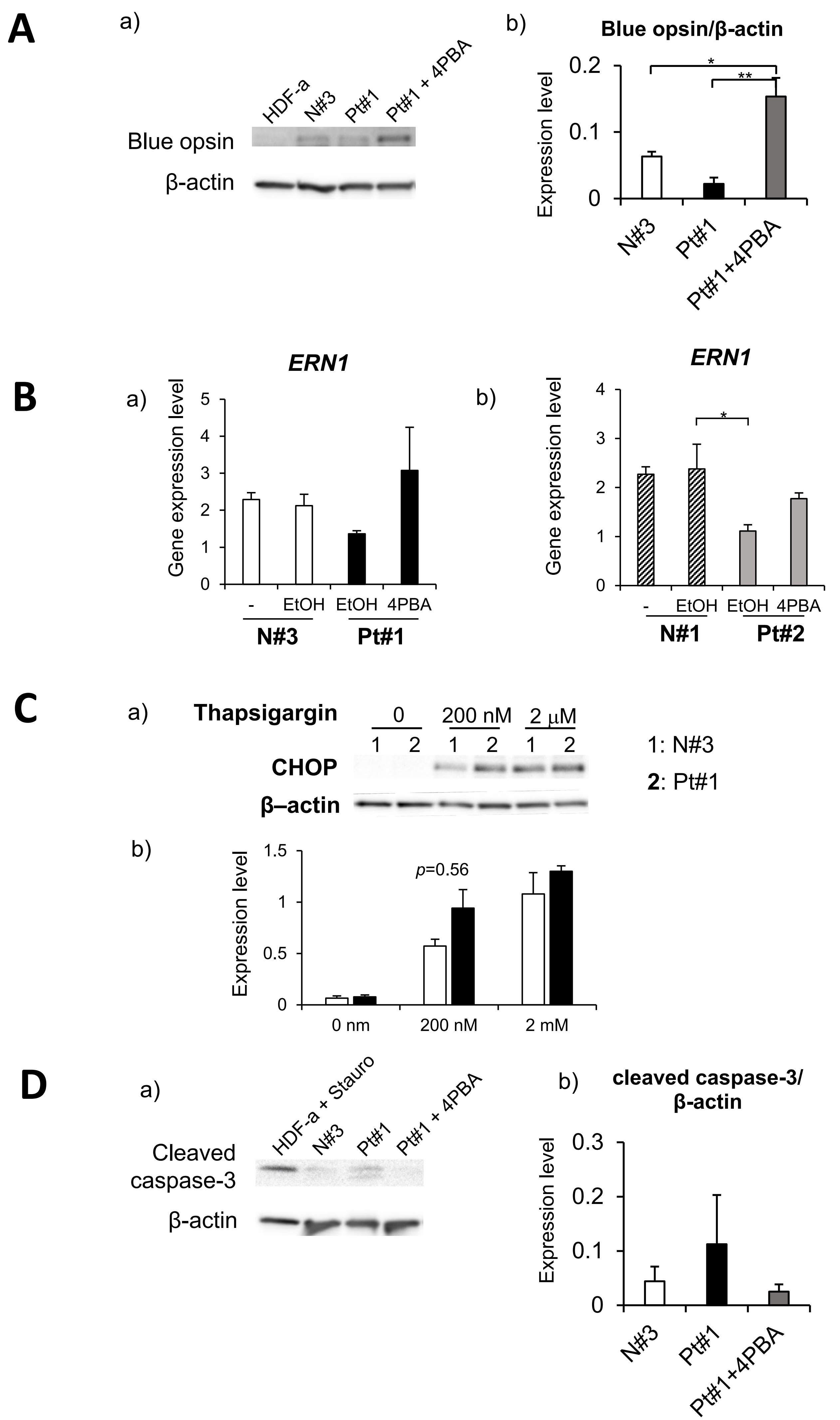

A
a)
HDF-a
N#3
Pt#1
Pt#1 + 4PBA
Blue opsin
β-actin
b)
Blue opsin/β-actin
Expression level
0.2
0.1
0
*
**
N#3
Pt#1
Pt#1+4PBA
B
ERN1
a)
Gene expression level
5
4
3
2
1
0
-
EtOH
EtOH
4PBA
N#3
Pt#1
ERN1
b)
Gene expression level
4
3
2
1
0
*
-
EtOH
EtOH
4PBA
N#1
Pt#2
C
a)
Thapsigargin
0
200 nM
2 μM
1 2 1 2 1 2
CHOP
β–actin
1: N#3
2: Pt#1
b)
Expression level
1.5
1
0.5
0
p=0.56
0 nm
200 nM
2 mM
D
a)
HDF-a + Stauro
N#3
Pt#1
Pt#1 + 4PBA
Cleaved caspase-3
β-actin
b)
cleaved caspase-3/ β-actin
Expression level
0.3
0.2
0.1
0
N#3
Pt#1
Pt#1+4PBA

Figure S4

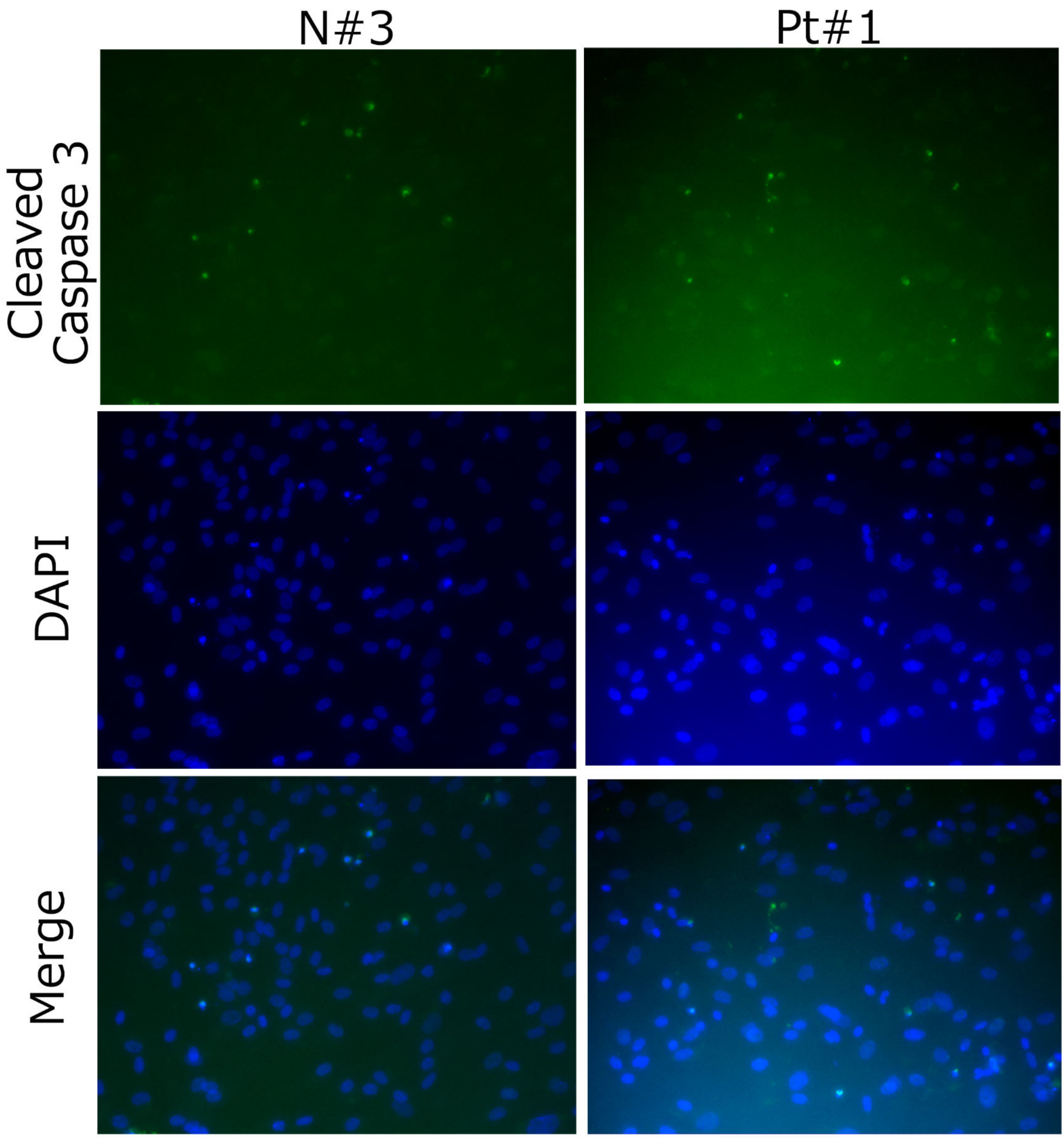

N#3

Thapsigargin (μM)	0.04	0.2	1
	7	3	14
total	187	129	306
%	3.743316	2.325581	4.575163
	19	5	21
total	123	112	196
%	15.44715	4.464286	10.71429
Ave	9.595235	3.394934	7.644725

Pt#1

Thapsigargin (μM)	0.04	0.2	1
	12	55	47
total	74	109	139
%	16.21622	50.45872	33.81295
	8	52	57
total	80	140	86
%	10	37.14286	66.27907
Ave	13.10811	43.80079	50.04601

Figure S5
